# *Tet3* regulates cellular identity and DNA methylation in neural progenitor cells

**DOI:** 10.1007/s00018-019-03335-7

**Published:** 2019-10-23

**Authors:** Mafalda Santiago, Claudia Antunes, Marta Guedes, Michelina Iacovino, Michael Kyba, Wolf Reik, Nuno Sousa, Luísa Pinto, Miguel R. Branco, C. Joana Marques

**Affiliations:** 1 Life and Health Sciences Research Institute (ICVS), School of Medicine, University of Minho, 4710-057 Braga, Portugal; 2 ICVS/3B’s—PT Government Associate Laboratory, 4710-057 Braga/Guimarães, Portugal; 3 Lillehei Heart Institute and Department of Pediatrics, University of Minnesota, Minneapolis, MN 55455 USA; 4 Epigenetics Programme, The Babraham Institute, Cambridge, CB22 3AT UK; 5 The Wellcome Trust Sanger Institute, Cambridge, CB10 1SA UK; 6 Blizard Institute, Barts and The London School of Medicine and Dentistry, Queen Mary University of London, London, E1 2AT UK; 7 Department of Genetics, Faculty of Medicine, University of Porto, 4200-319 Porto, Portugal; 8 i3S—Instituto de Investigação e Inovação em Saúde, Universidade do Porto, 4200-135 Porto, Portugal; 9 Present Address: Division of Medical Genetics, Department of Pediatrics, Harbor UCLA Medical Center, Los Angeles Biomedical Research Institute, Torrance, CA 90502 USA

**Keywords:** TET enzymes, 5-hydroxymethylcytosine, Imprinted genes, Neural stem cells, Pluripotency, Neurogenesis

## Abstract

TET enzymes oxidize 5-methylcytosine (5mC) into 5-hydroxymethylcytosine (5hmC), a process thought to be intermediary in an active DNA demethylation mechanism. Notably, 5hmC is highly abundant in the brain and in neuronal cells. Here, we interrogated the function of *Tet3* in neural precursor cells (NPCs), using a stable and inducible knockdown system and an in vitro neural differentiation protocol. We show that *Tet3* is upregulated during neural differentiation, whereas *Tet1* is downregulated. Surprisingly, *Tet3* knockdown led to a de-repression of pluripotency-associated genes such as *Oct4*, *Nanog* or *Tcl1*, with concomitant hypomethylation. Moreover, in *Tet3* knockdown NPCs, we observed the appearance of OCT4-positive cells forming cellular aggregates, suggesting de-differentiation of the cells. Notably, *Tet3* KD led to a genome-scale loss of DNA methylation and hypermethylation of a smaller number of CpGs that are located at neurogenesis-related genes and at imprinting control regions (ICRs) of *Peg10*, *Zrsr1* and *Mcts2* imprinted genes. Overall, our results suggest that TET3 is necessary to maintain silencing of pluripotency genes and consequently neural stem cell identity, possibly through regulation of DNA methylation levels in neural precursor cells.

**Electronic supplementary material:**

The online version of this article (10.1007/s00018-019-03335-7) contains supplementary material, which is available to authorized users.

## Introduction

DNA methylation, or 5-methylcytosine (5mC), is an epigenetic modification that consists of a methyl group added to the fifth position of cytosines, occurring more frequently in the context of CpG dinucleotides [1]. Albeit deemed as a very stable chemical modification, waves of global loss of DNA methylation occur during critical periods of development such as in the zygote and in primordial germ cells [2]. Additionally, loss of DNA methylation has been observed in post-mitotic cells, with activity-dependent demethylation occurring in mature neurons upon depolarization [3, 4]. This mechanism of active DNA demethylation remained elusive for a long time, but the finding that TET enzymes can convert 5mC into 5-hydroxymethylcytosine (5hmC), and subsequently into 5-formylcytosine (5fC) and 5-carboxylcytosine (5caC) [5–8], shed light into this mechanism. Importantly, 5hmC was shown to accumulate in the paternal pronucleus and in PGCs concomitantly with methylation loss [9–11] and to appear in an antagonistic way to 5mC in the genome of dentate granule neurons [12]. Three members—TET1, TET2 and TET3—compose the family of TET enzymes, which are Fe^2+^ and 2-oxoglutarate-dependent dioxygenases. TET1 and TET3 contain a CXXC zinc finger domain at their amino-terminus that is known to bind CpG sequences, whereas TET2 partners with IDAX, an independent CXXC-containing protein [13, 14]. 5hmC was first described in mouse embryonic stem (ES) cells and in Purkinje neurons [7, 8] and was later shown to be most abundant in the brain, namely at the cerebellum, cortex and hippocampus brain regions [15]. Moreover, TET enzymes were shown to be expressed in these brain regions, with *Tet3* showing highest expression [15]. Additionally, in the embryonic mouse brain, 5hmC levels were shown to increase during neuronal differentiation, as the cells migrate from the ventricular zone to the cortical plate [16]. In neurons, 5hmC was associated with gene bodies of activated neuronal function-related genes and gain of 5hmC was concomitant with loss of the repressive histone mark H3K27me3 [16]. Notably, TET enzymes have also been implicated in brain processes and functions such as neurogenesis, cognition and memory [17–21].

Here, we addressed the functional role of TET3 enzyme in neural precursor cells (NPCs) using an in vitro differentiation system, where highly proliferative ES cells are differentiated into a homogeneous population of NPCs that are PAX6-positive radial glial cells [22] and a stable and inducible RNAi knockdown system [23]. We observed that knockdown (KD) of *Tet3* in NPCs resulted in upregulation of pluripotency genes and genome-wide loss of DNA methylation. Nevertheless, gain of methylation was also observed, particularly in genes involved in neural differentiation. Our data suggest that TET3 plays a role in maintaining both cellular identity and DNA methylation levels in neural precursor cells.

## Results

### Neural differentiation leads to *Tet3* upregulation

**Fig. 1:**
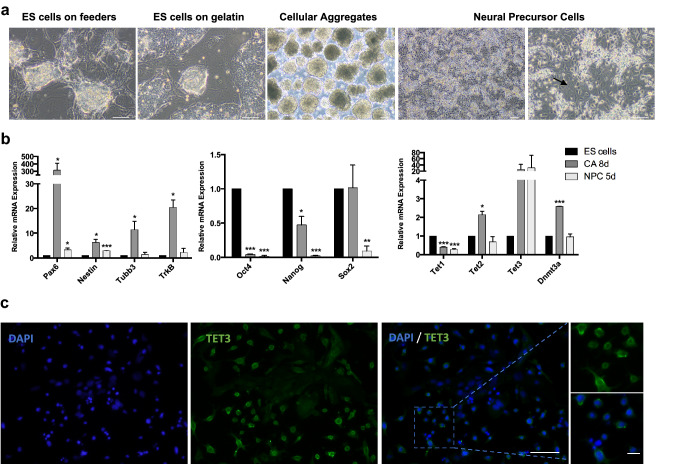
*Tet3* is upregulated during neural differentiation. **a** Neural differentiation protocol with representative images of key transition points—embryonic stem (ES) cells on feeders, ES cells on gelatin, cellular aggregates (CAs) and neural precursor cells (NPCs). Arrows show neurites forming between the cells; Scale bars—100 µm. **b** Relative expression of neural markers (*Pax6*, *Nestin*, *Tubb3* and *TrkB*), pluripotency markers (*Oct4*, *Nanog* and *Sox2*) and epigenetic regulators (*Tet1*, *Tet2*, *Tet3* and *Dnmt3a*) in several stages of the neural differentiation process—ES cells on gelatin (ES cells), CA after addition of Retinoic Acid (CA 8d), NPC after 5 days in culture (NPC 5d); *n* = 2 independent experiments; *p < 0.05; **p < 0.01; ***p < 0.001; *t* tests. **c** Immunostaining of TET3 in differentiated NPCs. Scale bars—100 µm and 25 µm

To investigate the effects of the knockdown of TET3 enzyme in NPCs, we established a stable and inducible knockdown system in mouse ES cells containing shRNAs targeting *Tet3* (Fig. S1a) [23, 24] and a neural differentiation system that results in a homogeneous population of PAX6-positive radial glial-like neural precursor cells (Fig. 1a, S1b, c) [25]. In this differentiation protocol, ES cells are maintained in a highly proliferative state and then cultured in non-adherent conditions forming cellular aggregates; addition of retinoic acid (RA) 4 days after cellular aggregates are formed results in upregulation of neural markers, such as *Pax6*, *Nestin*, *Tubb3* (B3-tubulin) and *TrkB* (*Nrtk2*) (Fig. 1b), with between 92 and 96% of the differentiated cell staining positively for PAX6 (Fig. S1b, c). This indicates homogeneous differentiation of ES cells into NPCs as described in the original protocol [22]. Positive staining of Beta 3-tubulin, which is one of the earliest markers of neuronal differentiation [26], was also observed (Fig. S1b). On the other hand, SOX2, which is a marker for neural stem cells that becomes inactivated in NPCs [27, 28], was nearly undetected (Fig. S1b). During differentiation, there was also a marked decrease in the expression of pluripotency genes such as *Oct4* and *Nanog*, as expected (Fig. 1b). Regarding epigenetic modifiers, we observed increased levels of *Tet3* and *Dnmt3a* during differentiation, whilst levels of *Tet1* decreased (Fig. 1b). Upregulation of *Tet3* during neuronal differentiation has been previously observed [29, 30] and suggests a prominent role for *Tet3* in the neuronal lineage. We also confirmed the presence of TET3 protein in NPCs by immunostaining, showing a predominantly cytoplasmic distribution (Fig. 1c); this is consistent with a putative role for TET3 in oxidizing 5mC to 5hmC in RNA molecules [31].

### Knockdown of *Tet3* in NPCs results in de-repression of pluripotency genes

**Fig. 2:**
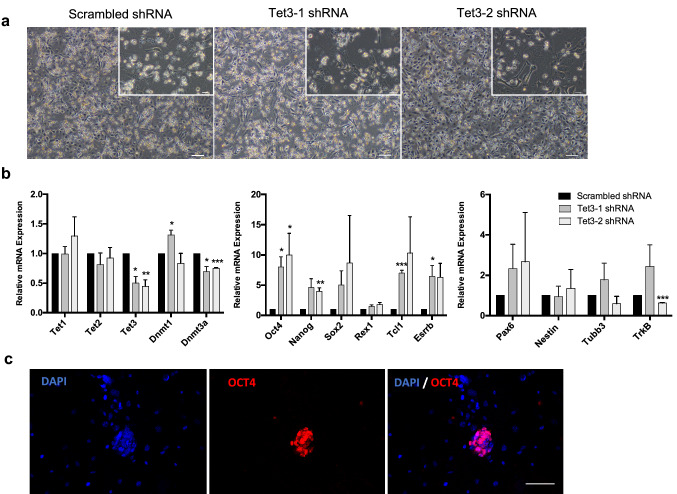
Knockdown of *Tet3* in NPCs results in de-repression of pluripotency genes. **a** Phase-contrast images of NPCs after Tet3 knockdown during 5 days in culture. Scrambled shRNA—control; Tet3-1 and Tet3-2 shRNAs—shRNAs against *Tet3*. Scale bars—100 µm and 50 µm in the insets. **b** mRNA transcript levels of epigenetic regulators (*Tet* and *Dnmt* enzymes), pluripotency genes (*Oct4*, *Nanog*, *Sox2*, *Rex1* and *Tcl1*) and neural markers [(stem cell markers—*Pax6* and *Nestin*; mature differentiation markers—B3-tubulin (*Tubb3*) and Neurotrophic tyrosine kinase, receptor, type 2 (*TrkB* or *Ntrk2*)] after *Tet3* knockdown. (*p < 0.05, **p < 0.01, ***p < 0.001; *t* test). Error bars represent SEM for three (Tet3-1 shRNA) and two (Tet3-2 shRNA) independent experiments. **c** Immunostaining of OCT4 in NPCs after *Tet3* KD, using Tet3-2 shRNA, shows OCT4-positive cells forming aggregates that resemble ES cell colonies. Scale bar—50 µm

We performed stable and inducible knockdown of *Tet3* in NPCs, using two independent shRNAs (Fig. 2a, b); *Tet3* knockdown was detected at both the mRNA and protein levels (Fig. 2b and S2a). Interestingly, we observed a significant upregulation of pluripotency genes, namely *Oct4, Nanog, Tcl1* and *Esrrb*, after *Tet3* KD (Fig. 2b), using two independent shRNAs. To further elucidate the observed upregulation of pluripotency genes, we performed immunostaining for OCT4 and observed the presence of OCT4-positive cells that appeared as cellular aggregates (Fig. 2c), representing around 14% of the total number of cells. Of note, OCT4-positive cells were not observed in NPCs treated with the Scrambled shRNA (Fig. S3); this suggests that Tet3 KD NPCs might have undergone a de-differentiation event due to downregulation of *Tet3* expression. This is in line with a recent report showing that *Tet3* can promote a rapid and efficient conversion of fibroblasts into neurons, showing that *Tet3* plays an important role in inducing and maintaining neural cell identity [32]. To better understand the nature of these ES cells like NPCs, we performed flow cytometry using Propidium iodide staining in KD NPCs and observed that *Tet3* KD NPCs still resemble control NPCs (Scrambled shRNA) more than ES cells, which show an extended S-phase comparing to NPCs (Fig. S2b). Additionally, we observed a significant increase in *Dnmt1* and decrease in *Dnmt3a* expression after *Tet3* KD (Fig. 2b), pointing to a co-regulation between TET enzymes and DNA methyltransferases.

These results suggest that functional perturbation of *Tet3* in NPCs leads to de-repression of pluripotency genes which might affect maintenance of the neural precursor cell identity.

### *Tet3* knockdown results in genome-scale loss of DNA methylation

As the above-mentioned results pointed to a critical role for *Tet3* in neural differentiation, we performed oxRRBS (oxidative Reduced Representation Bisulfite Sequencing) to analyse genome-wide changes in distribution of 5mC and 5hmC after *Tet3* knockdown. RRBS is a bisulfite-based protocol that enriches for CpG-rich parts of the genome, thereby reducing the amount of sequencing required, since it only covers 1% of the genome while capturing the majority of promoters and CpG islands [33]. To distinguish 5hmC from 5mC and since conventional sodium bisulphite treatment does not discriminate between the two modifications [34], we first added potassium perruthenate (KRuO_4_) that triggers selective chemical oxidation of 5hmC to 5-formylcytosine (5fC), before bisulphite treatment. 5fC is then further converted to uracil after bisulphite treatment and subtraction of oxidative bisulphite readout from the bisulphite—only one allows determining the amount of 5hmC at a particular nucleotide—in a single-base resolution and quantitative manner [35, 36]. As the bisulphite signal is always expected to be larger than that of oxidative bisulphite, negative values are artefacts used to estimate the false discovery rate (FDR; see Methods). Notably, we could only detect 2,191 hydroxymethylated CpGs (out of ~ 0.5 M) at a high FDR of 45% (Fig. S4a), which is in contrast with the low FDR (~ 3%) that we previously obtained in ES cells [35]. This is likely due to the fact that 5hmC levels are low in NPCs comparing to mouse ES cells and hippocampus brain region (Fig. S4b, c) [30] and mostly present in intragenic regions [16], whereas oxRRBS mainly captures promoters and CpG islands [33].

**Fig. 3:**
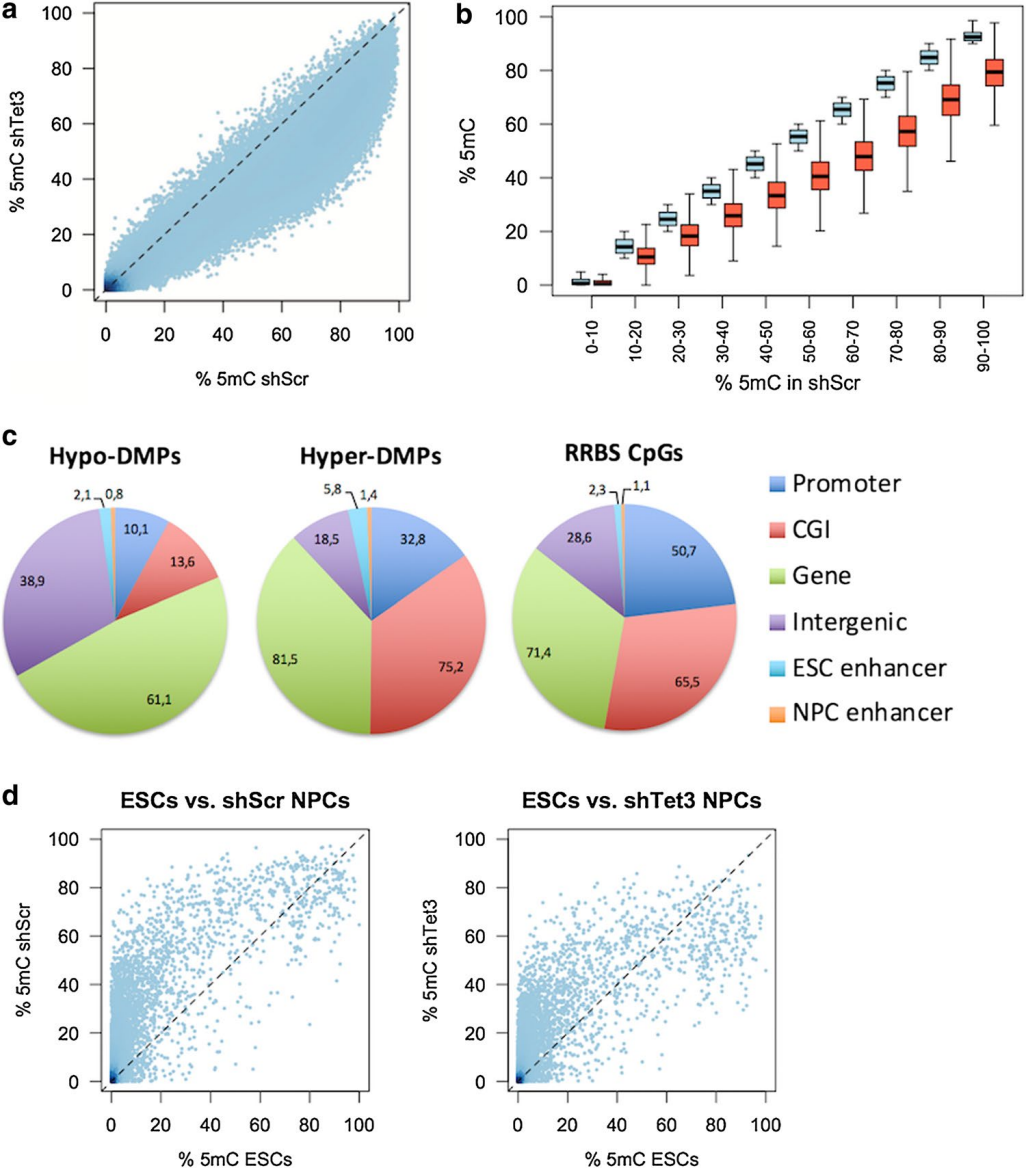
*Tet3* knockdown results in genome-scale loss of DNA methylation. **a** Scatter plot of 5mC levels at individual CpGs, showing a bulk shift in methylation after *Tet3* KD, using Tet3-2 shRNA. **b** To better visualize differences in 5mC levels, CpGs were grouped based on their  % 5mC in control NPCs. The plot displays the distributions of 5mC levels for control (blue) and *Tet3* KD (red) within each group. Loss of methylation is observed across the whole range of methylation levels. **c** Genomic features associated with differentially methylated positions (DMPs) after *Tet3* KD, showing that hypo-DMPs are enriched at genic regions and depleted at promoters and CpG islands. **d** Comparison of our oxRRBS datasets with a published dataset for ES cells [35], displaying average 5mC levels per CpG island

Notwithstanding, we observed an unexpected global loss of 5mC after *Tet3* KD (Fig. 3a, b). Loci showing loss of methylation covered the whole range of methylation levels, but particularly regions that had more than 40% of 5mC in control NPCs (Fig. 3b). We performed detection of differentially methylated positions (DMPs; *q* value < 0.01; > 10% difference), which yielded a total of 88,437 hypomethylated CpGs that were enriched at genic regions when compared to the distribution of CpGs captured by RRBS (Fig. 3c). In contrast, very few hypo-DMPs were located in promoters and CpG islands, which can be explained by the fact that these are already frequently devoid of methylation [1, 37]. On the other hand, we detected only 588 hypermethylated CpGs, which were mainly located at CpG islands and genic regions (Fig. 3c).

To investigate whether the hypomethylation pattern seen in *Tet3* knockdown NPCs resembles ES cells, we compared our NPC dataset to a previously published oxRRBS dataset on ES cells [35]. We first noted that many CpG islands in control NPCs displayed higher 5mC levels when compared to ES cells, whilst a group of CpG islands was highly methylated (> 70%) in both cell types (Fig. 3d). Upon *Tet3* KD, 5mC levels did become closer to those seen in ES cells, but only for lowly methylated CpG islands. Importantly, *Tet3* KD led to demethylation of highly methylated CpG islands, which does not match the ES cell profile (Fig. 3d). Were an ES cell subpopulation to be responsible for 5mC loss in *Tet3* KD NPC population, this would have led to maintenance of 5mC levels at highly methylated CpG islands. This prediction was confirmed by simulating 5mC patterns for cell mixtures of ES cells and NPCs, where increasing the proportion of ES cells only decreases the methylation at low-methylation CpG islands, whereas high-methylation CpG islands remain largely unchanged (Fig. S4e). These results suggest that the DNA hypomethylation observed in *Tet3* knockdown NPCs might reflect an epigenetic reprogramming event specific to the depletion of *Tet3* in NPCs.

### *Tet3* knockdown alters DNA methylation at developmentally relevant gene promoters

**Fig. 4:**
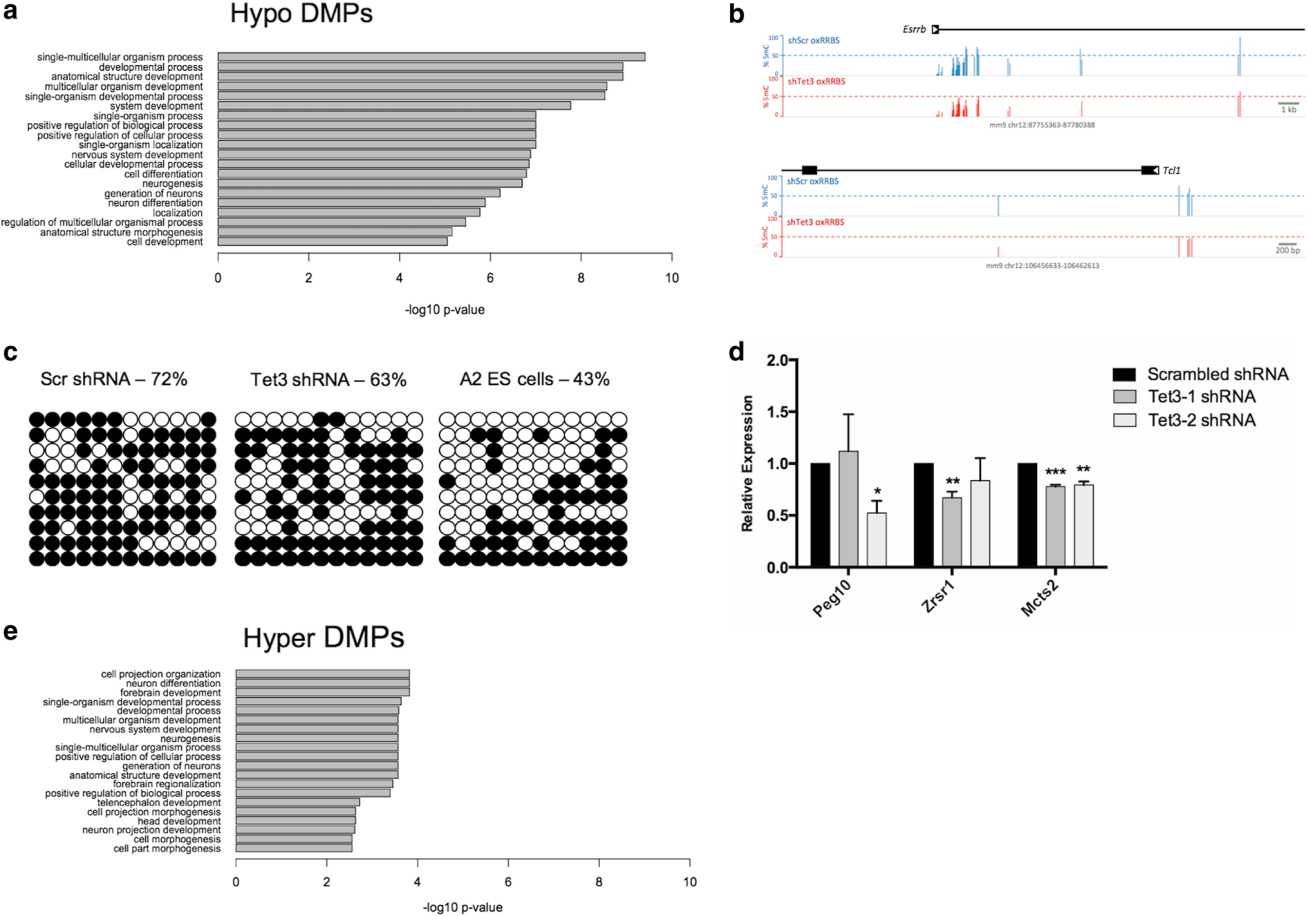
*Tet3* knockdown alters DNA methylation of developmentally relevant gene promoters. **a** Gene Ontology analysis of genes that loose methylation (Hypo DMPs) shows an association with development, differentiation and neurogenesis. **b** Genome browser snapshots of oxRRBS data at *Esrrb* and *Tcl1* pluripotency genes, showing a reduction in 5mC levels after *Tet3* KD. **c***Tcl1* bisulfite cloning analysis; black circles—methylated CpGs; white circles—unmethylated CpGs. **d** Expression analysis of imprinted genes showing hypermethylation after Tet3 KD (*p < 0.05; ***p < 0.001; *t* test); *n* = 2 independent experiments. **e** Gene Ontology analysis of genes that gain methylation (Hyper DMPs) shows an association with neural differentiation processes

To expand on these observations, we performed gene ontology analysis of genes associated with promoters harbouring groups of hypomethylated CpGs. For this purpose, differentially methylated regions (DMRs) were defined has regions showing at least 3DMPs with differences in the same direction. Promoters were defined − 1 kb to + 0.5 kb from mRNA TSSs. Promoters associated with hypomethylated DMRs (Supplemental file “Hyper_Hypo_promoters.xlsx”) were enriched for terms, such as development, differentiation and neurogenesis (Fig. 4a), suggesting that the observed hypomethylation is a regulated process coupled to the differentiation process between ES cells and NPCs. Of the genes involved in neurogenesis, *Slit1*, *Bdnf*, *Nr2e1* (*Tlx*), *Fgfr1*, *Runx1* and *Wnt3* are striking examples of genes that have been described to be involved in the proliferation of neural precursor cells [38–43]. Expression analysis of *Slit1* showed a tendency for increased mRNA transcription (Fig. S5), consistent with its hypomethylated state.

Moreover, loss of methylation was also observed at *Esrrb* and *Tcl1* early-pluripotency genes (Fig. 4b), which is in line with the observed upregulation of gene expression. Loss of methylation at *Tcl1* was confirmed by standard bisulfite sequencing (Fig. 4c).

For DNA hypermethylation, we only detected six genes with three or more hypermethylated CpGs at their promoters (Supplemental file “Hyper_Hypo_promoters.xlsx”). Notably, three of these genes are imprinted genes—*Peg10*, *Zrsr1* and *Mcts2*. Interestingly, it has been shown previously that loss of function of *Tet1* also leads to hypermethylation of imprinted genes, namely *Peg10* [44]. Expression analysis of these imprinted genes showed decreased expression in *Tet3* KD NPCs (Fig. 4d). More recently, it was also shown that *Tet3* regulates NSC maintenance through repression of *Snrpn* imprinted gene [45]. In accordance with this study, expression analysis of *Snrpn* in Tet3 KD NPCs showed increased transcription in one of the shRNAs (Fig. S5). To enable gene ontology analysis of hypermethylated sites, we changed our criteria to include promoters with a minimum of one hypermethylated CpG, yielding a total of 116 genes. Despite this low stringency, gene ontology analysis revealed significant associations with brain development, particularly with neuron differentiation and neurogenesis (Fig. 4e). Amongst these genes, *Wnt3a*, *Dlx2*, *Otx2* and *Rac3* are examples of genes described to promote neuronal differentiation [46–49], suggesting that TET3 plays a role in neurogenesis by maintaining hypomethylation of neuronal genes.

## Discussion

Several studies have previously addressed the role of TET1 in the brain, showing that it regulates processes such as memory and cognition, as well as expression of neuronal activity-regulated genes and hippocampal neurogenesis [17–19]. However, the role of TET3 in the nervous system remains largely unexplored. Here, we investigated the role of *Tet3* in NPCs, using a stable and inducible RNAi knockdown system and an in vitro neural differentiation protocol. Surprisingly, we observed that the knockdown of *Tet3* leads to de-repression of pluripotency genes and appearance of OCT4-positive aggregates of cells, suggesting that a reprogramming event is taking place in these cells. Indeed, when we analysed 5mC changes, we observed a dramatic genome-wide loss of methylation in *Tet3* KD NPCs. Hypomethylated CpGs were localized in genes involved in development, differentiation and neurogenesis. Loss of methylation was also observed in *Tcl1* and *Esrrb* pluripotency-associated genes suggesting a connection between loss of methylation, de-repression of pluripotency genes and de-differentiation of NPCs. A recent report on genome-wide DNA methylation in NPCs has shown an extensive demethylation from E18.5 NPCs relative to E11.5 NPCs, whereas only 1.5% of the identified DMRs gained methylation, suggesting that the acquisition of multipotency in E18.5 NPCs is associated with a wide loss of DNA methylation [50]. Furthermore, in mouse ES cells, it has been shown that *Tet2* knockdown results in both loss of 5hmC and 5mC at DMRs and promoters, while only few DMRs show the expected loss of 5hmC and gain of 5mC [51]. More recently, another study from the Rao lab reported that TET deficiency in diverse cell types resulted in localized increases in DNA methylation in active euchromatic regions, concurrently with unexpected losses of DNA methylation and reactivation of repeat elements [52].

Interestingly, we observed hypermethylation at three imprinted genes after *Tet3* knockdown. It had previously been shown that *Tet1* is necessary to induce 5mC oxidation at imprinting control regions (ICRs) of *H19/IGF2*, *PEG3* and *SNRPN/SNURF* imprinted genes, in a cell-fusion-mediated pluripotency reprogramming model [53]. Another study has shown that heterozygous offspring of *Tet1*/*Tet2* double knockout (DKO) mice show increased methylation levels across 94 ICRs, including *Peg10*, *Zrsr1* and *Mcts2* [54].

A critical role for *Tet3* in neural progenitor cell maintenance and terminal differentiation of neurons has been reported before [29]. As in our study, the authors observed an upregulation of *Tet3* upon neural differentiation and that *Tet3* KO in NPCs did not change expression of neural markers, such as *Pax6* and *Nestin*. Here, we also observed that neural markers are not altered, but pluripotency markers are de-repressed in *Tet3* KD NPCs, which suggests that the cells undergo de-differentiation upon downregulation of *Tet3* expression. We also observed that *Tet3* KD NPCs undergo a genome-scale loss of methylation, which is in contrast to what would be expected considering this enzyme as a demethylating agent. Indeed, we also observed hypermethylation, but in a more restricted number of sites, which are preferentially located in neuronal-related genes. The observed loss of methylation could potentially be caused by the concomitant decrease in *Dnmt3a* expression, which is a de novo methyltransferase playing a pivotal role in the nervous system [55, 56]. In fact, a functional interplay between TET1 and DNMT3A was shown in mouse embryonic stem cells [57]. Another interesting and perhaps more plausible explanation for the observed global demethylation might resides in the fact that TET enzymes might actually function as guides for de novo DNA methylation [58, 59]. In this context, it was reported that, in zygotes, *Tet3* might have a function in targeting de novo methylation activities, whereby *Tet3*-driven hydroxylation is predominantly implicated in the protection of the newly acquired hypomethylated state from accumulating new DNA methylation [58].

Intriguingly, Hahn and collaborators reported that functional perturbation of *Tet2* and *Tet3* in the embryonic cortex led to defects in neuronal differentiation with abnormal accumulation of cell clusters along the radial axis in the intermediate and ventricular zones [16]. Clustered cells did not express neuronal marker *B3*-*Tubulin* and some of the cells showed expression of *Nestin* in their processes, suggesting a defect in the progression of differentiation. This is in line with our observation that *Tet3* KD NPCs form clusters of cells that resemble ES-colonies and are OCT4-positive. Additionally, TET3 has been implicated in regulation of synaptic transmission [60, 61] and fear-extinction memory [21], which suggests a pivotal role in the nervous system.

In conclusion, our findings suggest that TET3 acts as a regulator of neural cell identity by maintaining DNA methylation levels in neural precursor cells.

## Experimental procedures

### Embryonic stem cell culture and neural differentiation

A2lox.cre mouse embryonic stem cells [23], were expanded on feeder cells (SNL767 feeder cell line, kindly provided by the Wellcome Trust Sanger Institute, UK) in complete ES medium–DMEM (4500 mg/L glucose; Gibco) supplemented with 110 mg/L sodium pyruvate (Gibco), 2 mM l-Glutamine (Gibco), 15% fetal bovine serum (Gibco, ES-cell tested), 1 × penicillin/streptomycin (Gibco), 0.1 mM MEM non-essential amino acids (Gibco) and 10^3^ U/ml LIF (ESGRO Millipore).

Neural differentiation of embryonic stem cells was performed as previously described [25]. Briefly, A2lox.cre ES cells (passage 17) containing shRNAs for *Tet3* were cultured on feeders for three passages and on 0.2% gelatine (Sigma) for another three passages. Subsequently, 4 × 10^6^ cells were plated onto bacterial non-adherent dishes (Greiner) for formation of non-adherent cellular aggregates (CA) in CA medium (DMEM 4500 mg/L glucose supplemented with 110 mg/L sodium pyruvate, 2 mM l-Glutamine, 10% fetal bovine serum, 1× penicillin/streptomycin and 0.1 mM MEM non-essential amino acids). CA medium was changed every other day and 5 μM of retinoic acid (RA; Sigma) was added from day 4 to day 8. CAs were then dissociated with freshly prepared Trypsin 0.05% (Sigma, powder) in 0.05% EDTA/PBS and plated onto Poly-DL-Ornithine and laminin-coated plates in N2 medium [DMEM/F12/Glutamax medium supplemented with 1x Penicillin–Streptomycin, 1× N2 supplement (Gibco) and 50 µg/mL BSA (Sigma)]. After 2 days, the medium was changed to a complete medium (N2B27: Neurobasal medium (Gibco), supplemented with 1× GlutaMAX (Gibco), 1x Penicillin–Streptomycin, 1× N2 supplement, 1x N2B27 supplement (Gibco)).

### Stable and inducible knockdown system

We used a stable and inducible knockdown system previously described by Iacovino and collaborators [23]. Briefly, shRNA-mir cassettes for *Tet3* gene (sequences on supplementary Table S1) were amplified from pSM2 retroviral vectors containing the shRNAmir sequences (Open Biosystems) and cloned into the p2Lox vector using HindII and NotI restriction sites. The p2Lox derivatives were transfected into the A2lox.cre ES cells (derived from the E14 male cell line strain 129P2/OlaHsd) expressing Cre after addition of doxycycline (0.5 μg/ml) to the medium 1 day before transfection. ES cells were transfected using Lipofectamine 2000 (Invitrogen) at a concentration of 5 × 10^5^ cells/ml. One day after transfection, selection medium containing geneticin (G418, Melford—300 μg/ml active concentration) was added to the cells during 10 days. After selection, ES cell clones containing the shRNAmir were expanded in ES complete medium and neural differentiation was performed as described above. For shRNA expression, doxycycline (2 μg/ml) was added to the medium during 5 days. An ES clone containing eGFP was used to control for positive induction after doxycycline addition. After these 5 days, the cells were trypsinized and the pellet was stored at − 80 °C until DNA/RNA/Protein extraction.

### Quantitative reverse transcription PCR

RNA was extracted using the AllPrep DNA/RNA mini kit (Qiagen) and cDNA was synthesized from 200 ng of RNA using the qScript cDNA Supermix (Quanta Biosciences). cDNA was diluted 1:10 and used as template for quantitative real-time PCR reactions using the 5x HOT FIREPol EvaGreen qPCR supermix (Solis Biodyne) and primers designed to specifically amplify each gene of interest (Supplementary Table S2). Cycling reactions were performed in duplicate and cycle threshold (Ct) fluorescence data recorded on Applied Biosystems 7500 Fast Real-time PCR System. The relative abundance of each gene of interest was calculated on the basis of the Delta Delta Ct method [62], where results were normalized to two housekeeping genes (*Atp5b* and *Hsp90ab1*). Statistical analysis was performed by multiple t tests using GraphPad Prism version 6.0 for Mac (GraphPad Software, La Jolla, CA, USA).

### Immunofluorescence microscopy and image analysis

Antibody staining of DNA methylation and hydroxymethylation was performed as previously described [24], with few modifications. Briefly, neural precursor cells were plated on glass coverslips and fixed with 2% paraformaldehyde for 30 min at room temperature (RT). Cells were permeabilised with phosphate-buffered saline (PBS) 0.5% Triton X-100 and treated with 2 N HCl for 30 min at RT. The coverslips were washed in PBS 0.05% Tween-20 (PBST) and blocked overnight in PBST with 1% bovine serum albumin (BSA) (BS). Cells were incubated with both primary antibodies rabbit anti-5hmC (1:500, Active Motif, 39792) and mouse anti-5mC (1:250, Eurogentec, BS-Mecy-0100) for 1 h at RT. For antibody staining of pluripotency and neuronal markers, cells were incubated with blocking buffer (BS) for 1 h at RT before incubation with primary antibodies overnight at 4 °C. Primary antibodies were rabbit anti-PAX6 (1:250, Millipore, AB2237), mouse anti-NESTIN (1:200, Millipore MAB353), rabbit anti-OCT4 (1:750, Abcam, ab18976), rabbit anti-SOX2 (1:1000, Abcam, ab97959), mouse anti-beta III tubulin (1:100, Millipore, MAB1637) and rabbit anti-TET3 (1:100, Abcam, 139805). After washing with BS for 1 h at RT, primary antibody staining was revealed with appropriate Alexa-Fluor-conjugated secondary antibodies (1:500, Molecular Probes). For both procedures, the nuclei were counterstained with DAPI. After washing with PBST, cells were mounted with Immu-mount (Thermo Scientific). Images were acquired on an Olympus BX61 or Olympus FV1000 (Japan) confocal microscope and analysed using ImageJ software^®^.

### Western blot for detection of TET3

Protein was extracted using the AllPrep DNA/RNA mini kit (Qiagen) and resuspended in 5% SDS. The protein concentration of the supernatants was determined using BCA kit (Pierce). Total lysates of 14 μg of protein were denatured in NuPage LDS sample buffer and NuPage reducing reagent by heating for 10 min at 95 °C. Proteins were separated on NuPage 4–12% Bis–Tris gels using MOPS running buffer (Thermofisher). Wet transfer onto a nitrocelulose membrane (Amersham Biosciences) was performed using MOPS running buffer with 20% methanol. Membranes were blocked with 10% milk/1% BSA in Tris-buffered saline (TBS)/0.1%Tween (TBS-T) overnight at 4 °C. Primary antibodies mouse anti-TET3 (1:1000, Abcam, ab174862) and mouse anti-α-Tubulin (1:5000, Sigma-Aldrich, T6074) diluted in blocking buffer and incubated 2 h at RT. Membranes were washed in TBS/T and incubated with the secondary antibody coupled to horseradish peroxidase (BioRad) 1 h at RT. The bound antibodies were visualized by chemiluminescence using ImageQuant LAS4000 mini (GE Healthcare). Bands were analysed using ChemiDoc (Bio-Rad) and quantification was performed with ImageLab software (Bio-Rad). α-Tubulin was used as loading control.

### Dotblot and ELISA analysis of 5hmC

DNA was extracted using the AllPrep DNA/RNA mini kit (Qiagen). Genomic DNA (100 ng) was denatured at 99 °C for 5 min and spotted on nitrocellulose blotting membranes (Amersham Hybond-N+). The membrane was UV-crosslinked for 2 min and then blocked in 10% milk/1% BSA in PBST overnight at 4 °C. The membranes were then incubated with rabbit anti-5hmC (1:500, Active Motif, 39769) for 1 h at RT. After washes with PBST (PBS 0.1% Tween-20), membranes were incubated with 1:10,000 dilution of HRP-conjugated anti-rabbit, washed with PBST and then treated with Amersham ECL (GE Healthcare). Dot blot intensities were analysed using ChemiDoc (Bio-Rad) and quantification was performed with ImageLab software (Bio-Rad).

The global level of 5-hmC was also assessed using Quest 5-hmC DNA ELISA Kit (Zymo Research). The procedure was followed according to the manufacturer’s instructions, loading 100 ng of DNA per well.

### Cell cycle analysis using flow cytometry for propidium iodide staining

For cell cycle analysis, NPCs were dissociated with Accutase (Sigma-Aldrich) for 10 min and re-suspended in 70% ethanol and kept at − 20 °C for 24 h for fixation. After fixation, cells were washed in 1× PBS and incubated with PI staining solution—Propidium Iodide 20 μg/ml (eBioscience) in PBS/0.1% Triton-X 100 and RNase 0.25 mg/ml (Invitrogen)—for 1 h at room temperature in the dark. Cell staining was then analysed by flow cytometry in a BD LSRII flow cytometer (BD Biosciences; 20,000 events). Analysis of the cell cycle was performed with ModFit LT (Verity Software House).

### Genome-wide analysis of DNA methylation and hydroxymethylation by oxRRBS

Genomic DNA was isolated using the Qiagen AllPrep DNA/RNA Mini kit (Qiagen) following manufacturers’ instructions. Oxidative Reduced Representation Bisulfite Sequencing (oxRRBS) was used for genome-wide analysis of DNA methylation and hydroxymethylation. This method relies on oxidation of DNA prior to bisulfite treatment to convert 5-hydroxymethylcytosine (5hmC) into 5-formylcytosine (5fC) which in turn will be converted to uracil (thymine after PCR amplification) (Fig. 4). 5-methylcytosine (5mC) remains unchanged after oxidation and bisulfite treatment and unmethylated cytosines will be converted to uracil (thymine after PCR amplification). By subtracting the two libraries, it is then possible to infer 5mC and 5hmC levels at a single-base resolution and in a quantitative manner [35].

Briefly, 100 ng of DNA were digested with MspI restriction enzyme and the reaction was cleaned up with AMPure XP beads (Agencourt). A library was then prepared with the NEBNext Ultra DNA library Prep for Illumina (NEB) for End repair, A-tailing and ligation of methylated adaptors (NEBNext, E7535), according to manufacturer’s’ instructions. Oxidation of the DNA was then carried out starting by purifying DNA in a Micro Bio-Spin column (BioRad), denaturing DNA with NaOH and adding 2 μL of Potassium Perruthenate (KRuO4, Alfa Aeser) solution (15 mM in 0.05 M NaOH). The reaction was held on ice for 1 h, purified with Micro Bio-Spin column (BioRad) and subjected to bisulfite treatment using the Qiagen Epitect kit, according to the manufacturer’s instructions for FFPE samples, except that the thermal cycle was run twice over. Final library amplification (18 cycles) was performed using Pfu Turbo Cx (Agilent) and adaptor-specific primers (barcoded TruSeq primers, Illumina), after which the libraries were purified using AMPure XP beads (Agencourt). To check for oxidation success, a spike-in control was added before oxidation step and amplified and digested with TaqI restriction enzyme at the end of library amplification.

### Sequencing and data processing

Sequencing (single-end, 75 bp reads) was performed on the Illumina NextSeq platform, high-throughput mode. Quality control of sequencing reads was performed with FASTQC (Babraham Bioinformatics). Trimming of the reads to remove adaptors and low-quality bases was performed using Trim-Galore with –rrbs option (Babraham Bioinformatics). The alignment was performed using Bismark with bowtie2 and methylation extraction with the options -s –comprehensive [63]. SeqMonk (Babraham Bioinformatics) and the R-package Methylkit [64] were used for downstream analysis.

DMPs were detected using the Methylkit [64]. We overlapped DMPs with genomic features. Promoters were defined − 1 kb to + 0.5 kb from mRNA TSSs (and deduplicated if > 50% overlapped), CpG islands are from Illingworth et al. [65] and enhancers are from ChIA-PET data [66]. Gene ontology analyses were performed using the topGO R package, focusing on biological process terms.

All sequencing data are available under Gene Expression Omnibus (GEO) accession number GSE123110.

### Gene-specific methylation levels by standard bisulfite sequencing

Genomic DNA was isolated using the AllPrep DNA/RNA Minikit (Qiagen) following manufacturers’ instructions. Five hundred nanograms of DNA were subjected to bisulfite treatment using the Epitect Bisulfite Kit (Qiagen). A CpG island on intron 1 of *Tcl1* gene (chromosome position 12:106,460,347–106,460,634, NCBI37 (mm9) mouse reference genome) was amplified using primers described in supplementary table S2 and HostStar MasterMix (Qiagen) with the following cycling conditions: 95 °C for 15 min followed by 35 cycles of 95 °C for 1 min, 58 °C for 1 min and 72 °C for 1 min, with a final extension of 72 °C for 20 min. PCR products were then cloned using the TOPO TA Cloning kit for sequencing (Invitrogen) and NZYalpha competent cells (NZYtech). Ten clones for each sample were picked and plasmid DNA amplified using M13 primers. PCR products for each clone were sequenced using the BigDye Terminator v3.1 cycle sequencing kit (Applied Biosystems) in an ABI 3500 Genetic Analyzer (Applied Biosystems). Only clones with more than 95% non-CpG cytosines converted were considered for the analysis, using BiQ Analyzer Software [67].

### Electronic supplementary material

Below is the link to the electronic supplementary material.

Supplementary material 1 (XLSX 305 kb)

Supplementary material 2 (DOCX 9291 kb)
