# Supplementary material for: Tet3 regulates cellular identity and DNA methylation in neural progenitor cells
Source: Cell Mol Life Sci. 2019 Oct 23;77(14):2871–83. doi: 10.1007/s00018-019-03335-7 (PMC7326798; doi:10.1007/s00018-019-03335-7)
Supplement: Supplementary file 2 — Supplementary material 2 (DOCX 9291 kb) [file 18_2019_3335_MOESM2_ESM.docx]

**a**

**c**

**b**

**Figure S1 - Stable and inducible systems for *Tet1* and *Tet3* knockdown in NPCs (related to Figure 1).**

(a) Schematic representation of the stable and inducible knockdown system using p2Lox as a vector containing the shRNAmir cassette that is transfected in A2lox.cre ES cells containing a tetracycline inducible element and pLox sites for site-specific recombination.

(b) Immunostaining for PAX6, B3-tubulin and SOX2, in NPCs after 1 day in culture in N2 medium (scale bars – 50 µm).

(c) Percentage of PAX6-positive NPCs in clones containing shRNAs and eGFP as control.

**b**

**a**

**Figure S2 - Knockdown of *Tet3* in Neural Progenitor Cells (related to Figure 2).**

(a) Western blot analysis of TET3 in KD in NPCs and quantification.

(b) Cell cycle analysis by flow cytometry, using propidium iodide (PI) staining, of NPCs after knockdown of Tet3 shows a lower S-phase in all NPCs comparing to ES cells.

Control NPCs - Scrambled shRNA (shScr); NPCs containing shRNAs against +against Tet3 (shTet3-1 and shTet3-2).

Scrambled shRNA


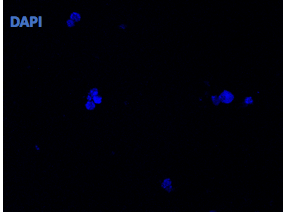

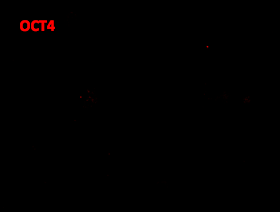

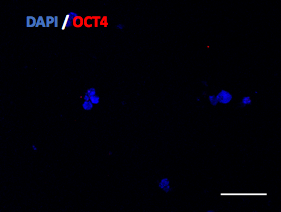


Tet3-2 shRNA


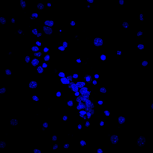

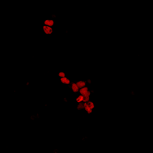

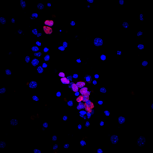

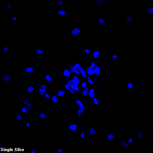

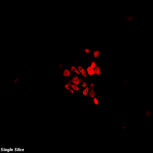

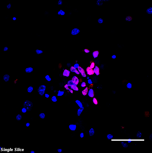

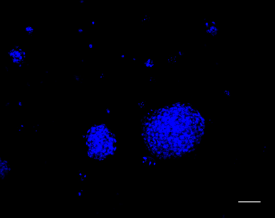

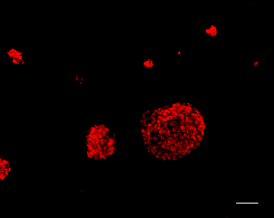

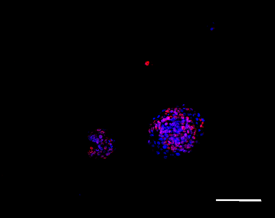


Mouse ES cells

**Figure S3 – OCT4 detection in Neural Precursor Cells (related to Figure 2)**

Immunostaining of OCT4 in NPCs showing OCT4-positive cells aggregating to form ES cell-like colonies after *Tet3* KD. shScr - Scrambled control shRNA; shTet3-2 - shRNA against Tet3; mES cells - mouse embryonic stem cells. Scale bars - 50 µm

**b**

ShTet3-2

Ctrl NPC

mES

mHipp

ShTet3-1

ShScr


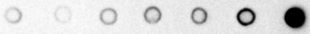

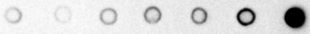


**a**

**c**

**d**


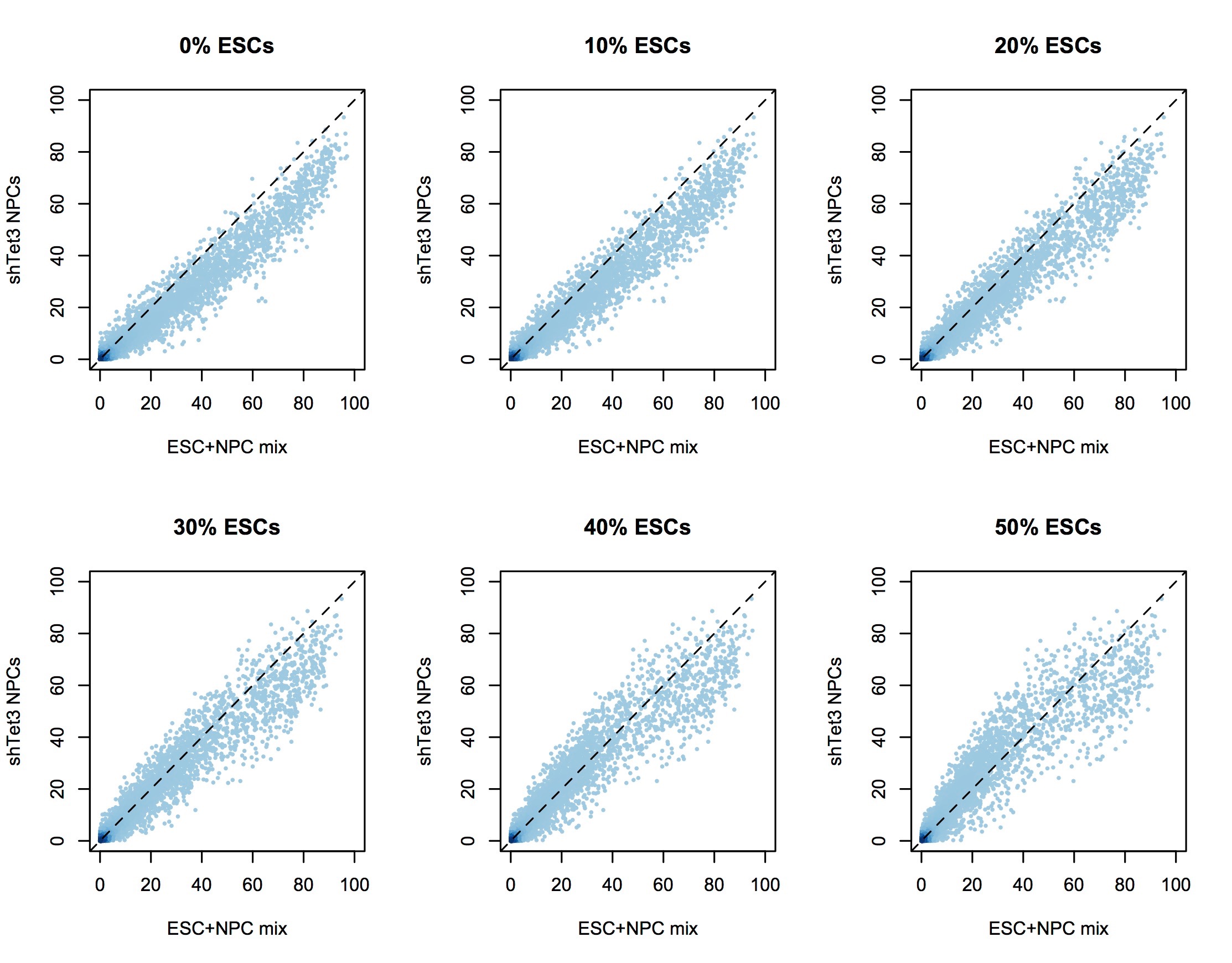


**e**

**Figure S4 - 5hmC and 5mC analysis in NPCs after *Tet3* KD (related to Figure 3).**

(a) oxRRBS data shows very little to no 5hmC signal in all the samples analysed

(b) Dot blot analysis of 5hmC in NPCs after *Tet3* KD. shScr - Scrambled control shRNA; shTet3-1 and shTet3-2 - shRNAs against *Tet3*. DNA from mouse ES cells (mES) and mouse hippocampal brain region (mHipp) were used as controls. 100 ng of DNA were loaded for all the samples.

(c) 5hmC detection by ELISA. DNA from mouse hippocampus (hipp1 and hipp2) was used as a positive control.

(d) Imunofluorescent detection of 5mC and 5hmC in NPCs shows that cells stain positively for both epigenetic marks. Scale bars – 50 µm.

(e) Comparison of oxRRBS methylation data between *Tet3* KD NPCs and different proportions of ES+NPC mixes.

**Figure S5 – Expression analysis of an hypomethylated gene (*Slit1*) and an imprinted gene (*Snrpn*).** (**p<0.01; t-test). Error bars represent SEM of three independent experiments for *Snrpn* and two independent experiments for *Slit1* (except Tet3-2 shRNA in which n=1 independent experiment).

**Supplementary Table S1. shRNAs sequences**

| **shRNA sequences** | |
| --- | --- |
| Scrambled (Scr) | tgctgttgacagtgagcgggtatattggaagcagaccttgtagtgaagccacagatgtacaaggtctgcttccaatatacctgcctactgcctcgga |
| Tet3-1 | tgctgttgacagtgagcgcgcccttgagctccaacgagaatagtgaagccacagatgtattctcgttggagctcaagggcatgcctactgcctcgga |
| Tet3-2 | tgctgttgacagtgagcgcgcagtgtgtattcctaccatttagtgaagccacagatgtaaatggtaggaatacacactgcttgcctactgcctcgga |
| Color codes: mir-30 context; sense; loop; anti-sense. | |

**Supplementary Table S2. Primer list and sequences**

|  | |  |  | **Product size (bp)** | Reference |
| --- | --- | --- | --- | --- | --- |
| **Bisulfite** |  |  |  |  |  |
| Tcl1  (12:106,460,347-106,460,634) | Fwd | AAATAGGAGGGTTAGGGAGATT |  | 288 |  |
|  |  |  |  |  |  |
|  | Rev | AAACACCAACATTAAAACCCA |  |  |  |
|  |  |  |  |  |  |
| **RT-qPCR** | |  |  |  |  |
| Atp5b | | GGCCAAGATGTCCTGCTGTT |  | 106 | Ficz *et al.*, 2011 |
|  |  | GCTGGTAGCCTACAGCAGAAGG |  |  |  |
| Hsp90ab1 | | GCTGGCTGAGGACAAGGAGA |  | 93 | Ficz *et al.*, 2011 |
|  |  | CGTCGGTTAGTGGAATCTTCATG |  |  |  |
| Tet1 | | CCATTCTCACAAGGACATTCACA |  | 116 | Ficz *et al.*, 2011 |
|  |  | GCAGGACGTGGAGTTGTTCA |  |  |  |
| Tet2 | | GCCATTCTCAGGAGTCACTGC |  | 120 | Ficz *et al.*, 2011 |
|  |  | ACTTCTCGATTGTCTTCTCTATTGAGG |  |  |  |
| Tet3 | | GGTCACAGCCTGCATGGACT |  | 104 |  |
|  |  | AGCGATTGTCTTCCTTGGTCAG |  |  |  |
| Dnmt1 | | TGTTCTGTCGTCTGCAACCT |  | 155 |  |
|  |  | CCCTCACACACTCCTTTTCTG |  |  |  |
| Dnmt3a | | CCTGCAATGACCTCTCCATT |  | 89 |  |
|  |  | CAGGAGGCGGTAGAACTCAA |  |  |  |
| Oct4 | | GAAGCCGACAACAATGAGAACC |  | 111 |  |
|  |  | CTCCAGACTCCACCTCACACG |  |  |  |
| Nanog | | CAGTGGTTGAAGACTAGCAATGGT |  | 113 |  |
|  |  | AGGCTTCCAGATGCGTTCAC |  |  |  |
| Sox2 | | GAGTGGAAACTTTTGTCCGAGA |  | 151 |  |
|  |  | GAAGCGTGTACTTATCCTTCTTCAT |  |  |  |
| Rex1 (Zfp42) | | CGATGCTGGAGTGTCCTCAAG |  | 113 | Ficz *et al.*, 2011 |
|  |  | GCCACACTCTGCACACACGT |  |  |  |
| Tcl1 | | CTCCATGTATTGGCAGATCCTGTA |  | 79 | Ficz *et al.*, 2011 |
|  |  | CTCCGAGTCTATCAGTTCAAGCAA |  |  |  |
| Esrrb | | AGTACAAGCGACGGCTGGAT |  | 103 | Ficz *et al.*, 2011 |
|  |  | CCTAGTAGATTCGAGACGATCTTAGTCA |  |  |  |
| Pax6 | | CAGATGCAAAAGTCCAGGTG |  | 209 | Bibel *et al.*, 2007 |
|  |  | TCTGTCTCGGATTTCCCAAG |  |  |  |
| Nestin | | TCGCTCAGATCCTGGAAGGTGG |  | 165 |  |
|  |  | GCTTCAGCTTGGGGTCAGGAAAG |  |  |  |
| Tubb3 | | GTGAAGTCAGCATGAGGGAGA |  | 195 |  |
|  |  | TGGGCACATACTTGTGAGAGGA |  |  |  |
| TrkB | | CTGGGGCTTATGCCTGCTG |  | 100 | Bibel *et al.*, 2007 |
|  |  | AGGCTCAGTACACCAAATCCTA |  |  |  |
| Gfap | | CGAAGAAAACCGCATCACCATTCC |  | 88 |  |
|  |  | TTGGCCTTCCCCTTCTTTGGTG |  |  |  |
| Peg10 | | AGAGCAGCCAACCGAGAAGGT |  | 164 |  |
|  |  | AACCCGCCTGTTCCACACGA |  |  |  |
| Zrsr1 | | ATGGTACGCAGGACGACAGC |  | 193 |  |
|  |  | AGTCCAAGCCGGAGGAGACAT |  |  |  |
| Mcts2 | | ACCCGTTTATCCTGCCACACC |  | 180 |  |
|  |  | TGACTCCGACACACAGGGCAT |  |  |  |
| Slit1 | | CAGGCTTTGGTGGCCCTGAATG |  | 125 |  |
|  |  | TGTGGAGACCTGAAGAGTGATGTT |  |  |  |
| Snrpn | | TGAGTTCAGGAAGATCAAGCCAAAG |  | 115 |  |
|  |  | GCCCTCCACAGTCATTGAAAC |  |  |  |
